# Effect of Simulated Geomagnetic Activity on Myocardial Ischemia/Reperfusion Injury in Rats

**DOI:** 10.21470/1678-9741-2018-0306

**Published:** 2019

**Authors:** Hui Wu, Weiyu Chang, Yanglin Deng, Xinli Chen, Yongli Ding, Xuesong Li, Liang Dong

**Affiliations:** 1 Department of Pharmacy, The First Affiliated Hospital of Kunming Medical University, Kunming, Yunnan, People’s Republic of China.; 2 Yunnan Observatories, Chinese Academy of Sciences, Kunming, Yunnan, People’s Republic of China.

**Keywords:** Hemodynamics, Myocardial Reperfusion Injury, Creatine Kinase, Troponin I, L-Lactate Dehydrogenase, Disease Outbreaks, Animal, Rats

## Abstract

**Objective:**

To study the response of myocardial ischemia/reperfusion injury (MI/RI) in rats to simulated geomagnetic activity.

**Methods:**

In a simulated strong geomagnetic outbreak, the MI/RI rat models were radiated, and their area of myocardial infarction, hemodynamic parameters, creatine kinase (CK), lactate dehydrogenase (LDH), melatonin, and troponin I values were measured after a 24-hour intervention.

**Results:**

Our analysis indicates that the concentrations of troponin I in the geomagnetic shielding+operation group were lower than in the radiation+operation group *(P*<0.05), the concentrations of melatonin in the shielding+operation group and normal+operation group were higher than in the radiation + operation group (*P*<0.01), and the concentrations of CK in the shielding + operation group were lower than in the radiation + operation group and normal + operation group (*P*<0.05). Left ventricular developed pressure (LVDP) and ± dP/dtmax in the radiation+operation group were lower than in the shielding + operation group and normal+operation group (*P*<0.01). Left ventricular end-diastolic pressure (LEVDP) in the shielding + operation group was higher than in the normal + operation group (*P*<0.05). There was no significant difference in area of myocardial infarction and LDH between the shielding + operation group and the radiation + operation group.

**Conclusion:**

Our data suggest that geomagnetic activity is important in regulating myocardial reperfusion injury. The geomagnetic shielding has a protective effect on myocardial injury, and the geomagnetic radiation is a risk factor for aggravating the cardiovascular and cerebrovascular diseases.

**Table:** 

Abbreviations, acronyms & symbols	
CK	= Creatine kinase
cTnI	= Troponin I
ELISA	= Enzyme-linked immunosorbent assay
LAD	= Left anterior descending
LDH	= Lactate dehydrogenase
LEVDP	= Left ventricular end-diastolic pressure
LVDP	= Left ventricular developed pressure
MI	= Myocardial ischemia
RI	= Reperfusion injury
SPSS	= Statistical Package for the Social Sciences
TTC	= Triphenyl tetrazolium chloride

## INTRODUCTION

The Earth's magnetic field is a protective barrier to life. The growth, development, and migration of living things require this magnetic field. Human activities are inextricably linked to the Earth's magnetic field as well. At present, about 3.5 million people die of cardiovascular and cerebrovascular diseases each year in China, accounting for over 40% of the total number of deaths due to various causes^[1]^. Studies have shown that the occurrence and development of cardiovascular and cerebrovascular diseases are closely related to the geomagnetic field, but the specific mechanism of influence is not yet clear^[2,3]^. The traditional research methods can't explain the mechanism of geomagnetic activity on cardiovascular and cerebrovascular diseases. Therefore, this project intends to simulate geomagnetic activity and study the response of myocardial ischemia/reperfusion injury (MI/RI) in rats to this geomagnetic activity. It will provide a new way to study the relationship between geomagnetic activities and cardiovascular diseases. And the results of this study can provide a new idea to treat the patients with MI/RI, when there is a geomagnetic outbreak.

## METHODS

### Animal and Ethical Statement

The present study used eight to ten-week-old male Sprague-Dawley rats (Charles River), weighing 200±20 g, which were obtained from the Experimental Animal Center of the Kunming Medical University (Kunming, Yunnan, China) and were housed in the Laboratory Animal Center of the Kunming Medical University. Animal experimental protocols were approved and performed according to the guidelines of the Institutional Medical Experimental Animal Care Committee of the Kunming Medical University. Guidelines for Laboratory Animal Care and Safety from the United States National Institutes of Health (Bethesda, Maryland, USA) were also followed.

### Reagents

Triphenyl tetrazolium chloride (TTC) was obtained from the Sigma-Aldrich Corporation (Missouri, USA). Kits for detecting lactate dehydrogenase (LDH), creatine kinase (CK), and troponin I (cTnI) were purchased from AU Clinical Chemistry Systems of the Beckman Coulter Inc. company (California, USA). The kit for detecting melatonin was purchased from the Abcam Inc. company (ab213978, Abcam, Cambridge, Massachusetts, USA).

### Equipment

**Fig. 1:**
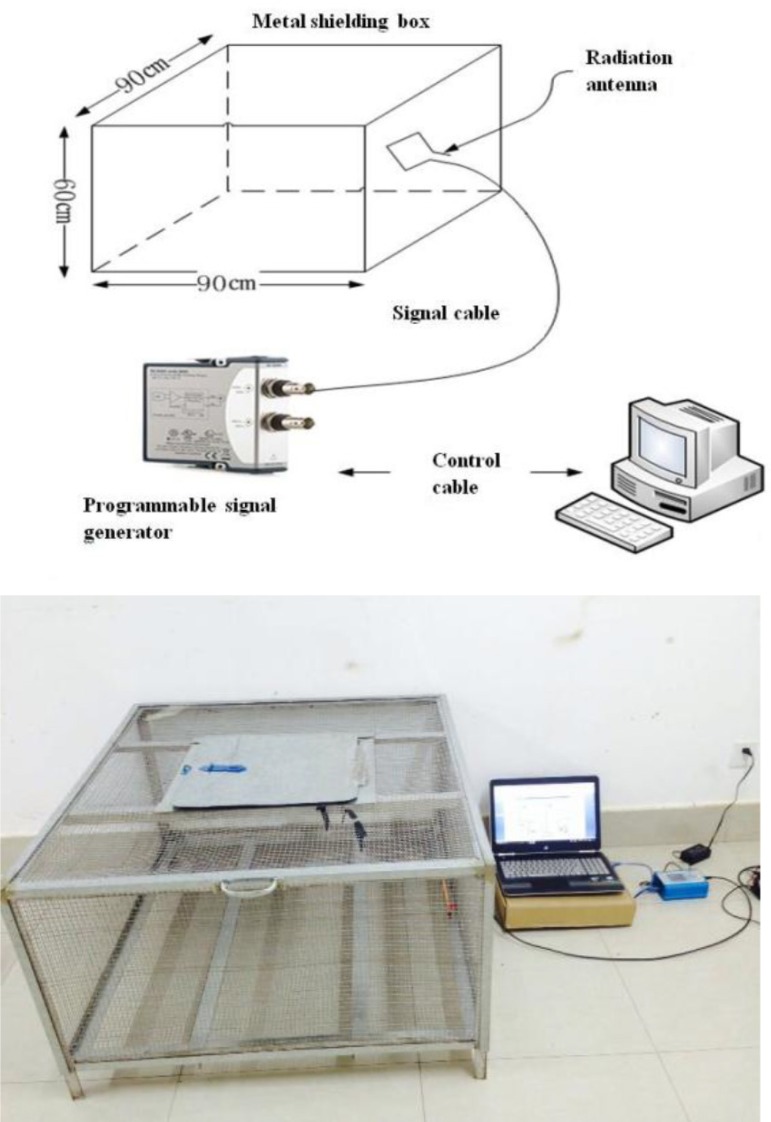
The structure drawing of geomagnetic experimental platform.

The geomagnetic experimental platform consists of four partial compositions, including a metal shielding experiment cage, radiation antenna, programmable signal generator, and control computer. The signal generator controlled by the main control computer can produce below 50 Hz and an arbitrary combination of signal spectra, especially analog Schumann resonances and geomagnetic burst, in the space of electromagnetic radiation (Figure 1). Patent No.: ZL201520208744.2.

### Animal Grouping and Treatment

The male rats were randomly divided into the geomagnetic shielding place, geomagnetic radiation place, and normal place (rats living in general environment without intervention). The rats in each place were divided into sham operation group and operation group, being nine rats in each group. The shield+sham group, radiation+sham group, normal+sham group, and normal+operation group were the control group. The shield+operation group and radiation+operation group were the experimental group.

### MI/RI Protocol

The MI/RI model was generated and utilized as previously described^[4,5]^. Rats were anesthetized using an intraperitoneal injection of 3.6% chloral hydrate (1 ml/kg) and ventilated isoflurane gas via tracheal intubation, with an RWD rodent respirator. Body temperature was maintained at 37ºC via a heated operating table. MI/RI surgery was performed using the following procedure: the heart was exteriorized by a left thoracic incision, and a slipknot (6-0 silk) was placed and ligated around the left anterior descending (LAD) coronary artery. The slipknot was released after 30 min of ischemia, and after that the animal received 24 hours of reperfusion. Ischemia was confirmed by noting the change in color of myocardial tissue in the ischemic area, and reperfusion was achieved by loosening the knot. The MI/RI rats were placed in different places for 24 hours of reperfusion, and then their physiological and biochemical indicators were tested.

### Myocardial Infarct Sizes

**Fig. 2:**
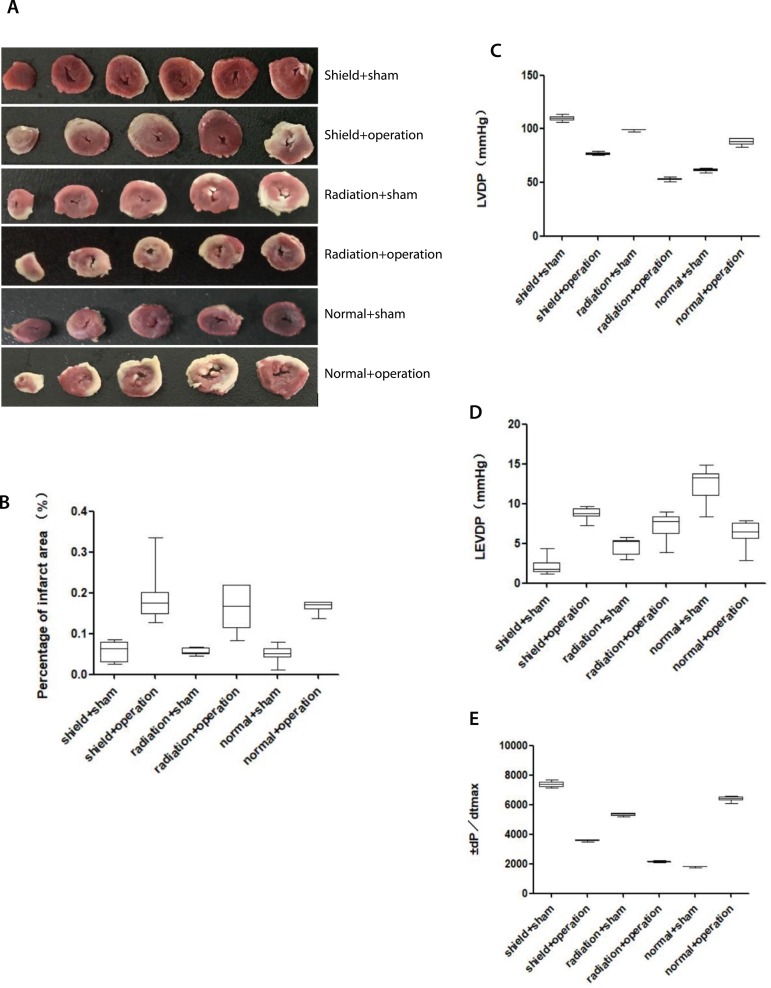
Heart physiological indexes. A) results of TTC staining of the myocardium of rats; B) The infarct areas of the left ventricular tissue were measured by TTC staining (n=6); C, D, and E) the left ventricular pressures were recorded by electrophysiolograph (n=9). LEVDP=left ventricular end-diastolic pressure; LVDP=left ventricular developed pressure; TTC=triphenyl tetrazolium chloride

Following reperfusion, the heart was then excised, both atria and the right ventricle were removed, and the left ventricle was cut into five equal slices to create cross sections from apex to base. The slices were separated into normal zone and area at risk, both followed by incubation in 1% TTC to measure the viability of myocardial tissue. Viable tissue stained red, while nonviable tissue remained unstained or was white (Figure 2A). Infarct size as a percentage of area at risk was determined gravimetrically.

### Left Ventricular Pressure Test

Cardiac function was measured by left ventricular cannulation. Rats were fasted for 12 hours and anesthetized with 3.6% chloral hydrate (1 ml/kg). The left common carotid artery was isolated, then the left ventricular developed pressure (LVDP), left ventricular end-diastolic pressure (LVEDP), and ± dP/dtmax were recorded by electrophysiolograph (BIOPAC 150).

### Serum Biochemistry Test

The blood was collected from the abdominal aorta artery catheter and added to procoagulant tube to centrifuge for 3000 min at 4ºC. The serum was stored in an ultra-low temperature refrigerator at -20ºC. The automatic blood biochemical analyzer (Beckman AU480, USA) detect cTnI, CK, and LDH in the blood with specific reagents from AU Clinical Chemistry Systems.

### Melatonin Test

The serum level of melatonin was detected by using the enzyme-linked immunosorbent assay (ELISA) kit according to the manufacturer’s instructions. The results were determined spectrophotometrically at 450 nm.

### Statistical Analysis

All statistical analyses were performed using the Statistical Package for the Social Sciences (SPSS) software, version 19 (SPSS Inc., Chicago, Illinois, USA). The data were presented as MEDIAN ± INTERQUARTILE RANGE and were compared using Kruskal-Wallis test. *P*<0.05 was considered statistically significant.

## RESULTS

### Myocardial Infarction Area

The results showed that there was no significant difference in the myocardial infarction area between the shielding + operation group, radiation + operation group, and normal+operation group (*P*>0.05), but there was significant difference in infarct size between the sham group and operation group (*P*<0.05) (Figure 2B).

### Left Ventricular Pressure

The results showed that LVDP in the shield+operation group and normal+operation group were higher than in the radiation+operation group (*P*<0.05) (Figure 2C). There was no significant difference in LEVDP in the shielding+operation group and radiation+operation group (*P*>0.05), but in the shielding+operation group it was higher than in the normal+operation group (*P*<0.05) (Figure 2D). The ± dP/dtmax of the left ventricle in the shielding+operation group and normal+operation group were higher than that in the radiation+operation group(*P*<0.05) (Figure 2E).

### Troponin I

**Fig. 3:**
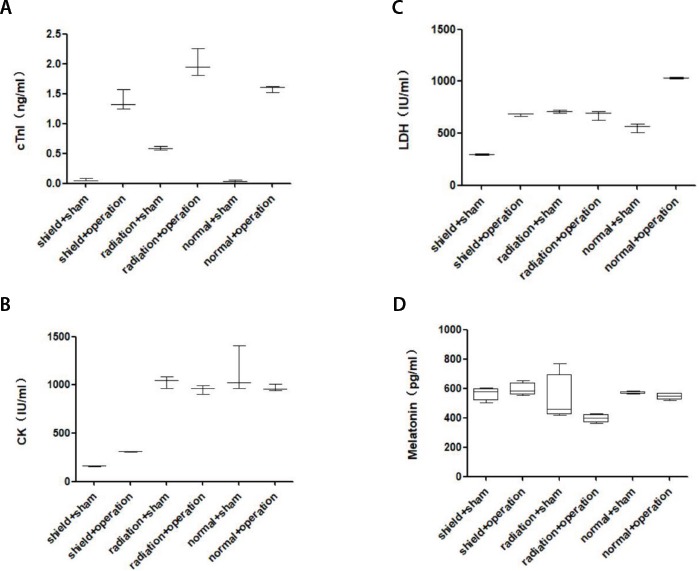
Serum biochemical indexes. A, B, and C). The cTnI, CK, and LDH were measured by automatic blood biochemical analyzer (n=3), which are specific indicators of myocardial infarction; D) melatonin was measured by ELISA kit, which protects the myocardial tissue (n=4). CK=creatine kinase; cTnI=troponin I; ELISA=enzyme-linked immunosorbent assay; LDH=lactate dehydrogenase

The results showed that cTnI in the radiation+operation group was higher than in the shielding+operation group, and in the operation group it was higher than in the sham group (*P*<0.05) (Figure 3A).

### Creatine Kinase

The results showed that CK in the shielding+operation group was lower than in the radiation+operation group and normal+operation group (*P*<0.05) (Figure 3B).

### Lactate Dehydrogenase

The results showed that LDH in the shielding+operation group had no significant difference from the radiation+operation group (*P*>0.05), but LDH in the shielding+operation group and radiation+operation group were lower than in the normal+operation group (*P*<0.05) (Figure 3C).

### Melatonin

The concentrations of melatonin in the radiation+operation group were significantly lower than in the shielding+operation group and normal+operation group (*P*<0.05) (Figure 3D).

## DISCUSSION

Among the many causes of MI/RI, humans began to notice the potential impact of geomagnetic activity on human health^[6,7]^. A vast number of large-scale clinical observational experiments show that when there was a geomagnetic outbreak, arrhythmia, hypertension, myocardial infarction, cerebral infarction, stroke, and other cardiovascular and cerebrovascular events increased significantly^[3,9-12]^. The mechanisms of the geomagnetic field on the cardiovascular function are studies that became very meaningful. Based on the discovered mechanism, we will prevent the geomagnetic effects and reduce the probability of cardiovascular diseases.

MI/RI lead to ventricular systolic and diastolic dysfunction^[13,14]^. From our experimental results, there was no significant difference on the myocardial infarction area between the shielding + operation group and radiation + operation group, but the LVDP and ± dP/dtmax in the radiation + operation group were lower than in the shielding + operation group and normal + operation group, and the LEVDP in the shielding + operation group was higher than in the normal + operation group, indicating that geomagnetic shielding can help to improve heart function and geomagnetic radiation can increase the damage in the heart function.

During MI/RI, myocardial oxygen free radicals accumulate, calcium overload occurs, and the myocardium produces a large amount of oxygen free radicals. These oxygen free radicals convert to hydroxyl free radicals, which act on the cell membrane and change the myocardial cell membrane structure and function. The cells release large amounts of LDH and CK, and they are specific indicators of myocardial injury^[14,15]^. From our experimental results, there are significant differences on CK between the shielding + operation group and radiation + operation group. This shows that geomagnetic activity has an impact on CK. We speculate that geomagnetic activity affects myocardial energy metabolism, muscle contraction, and adenosine triphosphate regeneration, because CK is correlated to those.

The cTnI is considered as the key biochemical marker in the diagnosis of myocardial injury. Patients with myocardial infarction have much higher concentrations of cTnI than healthy people^[16]^. From the experimental results, cTnI in the radiation + operation group is higher than in the shielding + operation group, indicating that geomagnetic radiation has an increasing effect on cTnI, while geomagnetic shielding has an inhibitory effect on cTnI, which proves that geomagnetic radiation has the potential to aggravate myocardial injury. Geomagnetic shielding has a protective effect on myocardial injury.

Melatonin has a powerful antioxidant effect and a high degree of diffusive penetration ability, which can exert its own antioxidant effect on the cell membrane, cytoplasm, and nucleus to protect the myocardial tissue against oxidative damage. From the experimental results, the concentration of melatonin in the radiation - operation group is lower than in the shielding + operation group and normal + operation group, indicating that geomagnetic radiation can reduce its protective effect on myocardial injury by inhibiting melatonin secretion. At the same time, the geomagnetic shield can promote the secretion of melatonin and enhance the protective effect of melatonin on myocardial injury^[12,14,16]^.

## CONCLUSION

The geomagnetic activity-related heat exposure is associated with an increase in cardiac events. This report describes the effect of geomagnetic outbreaks on the heart and how these outbreaks can aggravate damages from myocardial infarction. These findings identify the geomagnetic radiation damages to cardiovascular disease, which suggests that the impact of the space weather changing on human health cannot be ignored. In the view of the damage of geomagnetic outbreaks to the myocardium, we can predict the time of the geomagnetic outbreak in advance through early warnings from the astronomical observation, which may help to protect people from these outbreaks’ effects.

**Table:** 

Authors’ roles & responsibilities	
HW	Substantial contributions to the conception or design of the work; drafting the work or revising it critically for important intellectual content; final approval of the version to be published
WC	The acquisition, analysis, or interpretation of data for the work; final approval of the version to be published
YD	The acquisition, analysis, or interpretation of data for the work; final approval of the version to be published
XC	The acquisition, analysis, or interpretation of data for the work; final approval of the version to be published
YD	Final approval of the version to be published
XL	Substantial contributions to the conception or design of the work; drafting the work or revising it critically for important intellectual content; final approval of the version to be published
LD	is responsible for the commissioning of geomagnetic experimental platform; final approval of the version to be published

## Figures and Tables

**Table:** 

Geomagnetic shielding place		Geomagnetic radiation place		Normal place	
Shield+sham group	Shield+operation group	Radiation+sham group	Radiation+operation group	Normal+sham group	Normal+operation group
